# Assessment and elimination of the effects of head movement on MEG resting-state measures of oscillatory brain activity

**DOI:** 10.1016/j.neuroimage.2017.07.038

**Published:** 2017-10-01

**Authors:** Eirini Messaritaki, Loes Koelewijn, Diana C. Dima, Gemma M. Williams, Gavin Perry, Krish D. Singh

**Affiliations:** a Cardiff University Brain Research Imaging Centre (CUBRIC), School of Psychology, Cardiff University, Maindy Road, Cardiff, CF24 4HQ, UK; b BRAIN Unit, School of Medicine, Maindy Road, Cardiff University, Cardiff, CF24 4HQ, UK

**Keywords:** Magnetoencephalography (MEG), Resting-state analysis, Functional connectivity, Beamformer source localization

## Abstract

Magnetoencephalography (MEG) is increasingly being used to study brain function because of its excellent temporal resolution and its direct association with brain activity at the neuronal level. One possible cause of error in the analysis of MEG data comes from the fact that participants, even MEG-experienced ones, move their head in the MEG system. Head movement can cause source localization errors during the analysis of MEG data, which can result in the appearance of source variability that does not reflect brain activity. The MEG community places great importance in eliminating this source of possible errors as is evident, for example, by recent efforts to develop head casts that limit head movement in the MEG system. In this work we use software tools to identify, assess and eliminate from the analysis of MEG data any possible correlations between head movement in the MEG system and widely-used measures of brain activity derived from MEG resting-state recordings. The measures of brain activity we study are a) the Hilbert-transform derived amplitude envelope of the beamformer time series and b) functional networks; both measures derived by MEG resting-state recordings. Ten-minute MEG resting-state recordings were performed on healthy participants, with head position continuously recorded. The sources of the measured magnetic signals were localized via beamformer spatial filtering. Temporal independent component analysis was subsequently used to derive resting-state networks.

Significant correlations were observed between the beamformer envelope time series and head movement. The correlations were substantially reduced, and in some cases eliminated, after a participant-specific temporal high-pass filter was applied to those time series. Regressing the head movement metrics out of the beamformer envelope time series had an even stronger effect in reducing these correlations. Correlation trends were also observed between head movement and the activation time series of the default-mode and frontal networks. Regressing the head movement metrics out of the beamformer envelope time series completely eliminated these correlations. Additionally, applying the head movement correction resulted in changes in the network spatial maps for the visual and sensorimotor networks. Our results a) show that the results of MEG resting-state studies that use the above-mentioned analysis methods are confounded by head movement effects, b) suggest that regressing the head movement metrics out of the beamformer envelope time series is a necessary step to be added to these analyses, in order to eliminate the effect that head movement has on the amplitude envelope of beamformer time series and the network time series and c) highlight changes in the connectivity spatial maps when head movement correction is applied.

## Highlights

• Head movement effects on MEG resting-state measures of brain activity are evaluated.
• Regressing head movement metrics out of beamformer envelope time series is essential.
• Regressing head movement metrics out of network activation time series is essential.
• Regressing out head movement metrics changes some spatial network connectivity maps.

## Graphical abstract

**Figure:**
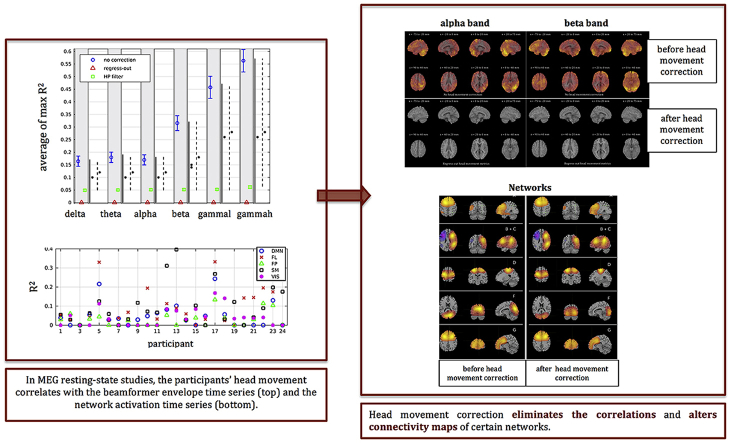

## 1 Introduction

Magnetoencephalography (MEG) is a neuroimaging technique that offers temporal precision of the order of milliseconds, provides good spatial resolution and is directly linked to neuronal activity, all of which render it well-suited to studies of brain function and of modulations of neuronal synchronization that are thought to underlie brain connectivity (Varela et al., 2001). MEG resting-state studies, namely studies during which MEG is recorded while participants are not engaged in any task, are widely used, especially when searching for differences in brain function between patient populations and control groups (Parisi et al., 2014, Zhao et al., 2012, Sanz-Arigita et al., 2010, Stam et al., 2006) and when trying to understand brain function in children and in elderly populations (Vlahou et al., 2014, Dimitriadis et al., 2013). This is largely due to the simplicity and short duration of resting-state recordings which make them non-intimidating to patients, children, and the elderly. It is also due to the lack of practice-related and performance-related confounds that can render task-based studies less reliable. Notwithstanding these important benefits, studying resting-state is justified by the documented correspondence between resting-state networks and task-related networks (Smith et al., 2009), which indicates that the fundamental organization of the human brain is relatively similar across task and resting-state conditions.

MEG recordings are performed with a spatially fixed sensor array, relative to which the participant's head can move. Any head movement during a MEG recording can result in source localization errors, which can be detrimental to the accuracy and statistical power of the analysis. A few studies of head movement in the MEG system have looked into such possible effects. Stolk and colleagues (Stolk et al., 2013) reported statistically significant effects of head movement on dipole reconstruction for MEG studies of somatosensory, visual and auditory tasks. They reported that offline incorporation of the head position time series into the general linear model results in improvements of group-level statistical sensitivity ranging between 15% and 29%. Uutela and colleagues (Uutela et al., 2001) studied the effect of head movement on one participant undergoing MEG recordings for an auditory paradigm. They tested two different head movement correction methods, a forward calculation correction method and a minimum-norm estimate correction method. Both methods significantly reduced the bias caused by the head movement of the participant and rendered it negligible compared to other sources of error in the study. Significantly, this work demonstrated that the effect of head movement is evident at the level of signal generation. Taulu and colleagues (Taulu and Kajola, 2005, Taulu et al., 2005) introduced the signal space separation method which, using a combination of two expansions of harmonic functions, can remove external disturbances from the signals and perform head movement corrections at the same time. The added advantage of the method is that it takes into account the inverse square distance nature of the signal. Wehner and colleagues (Wehner et al., 2008) studied head movement in children during an auditory paradigm and suggested ways to correct for the resulting errors in source estimation. However, head movement effects on MEG resting-state analyses have not, to our knowledge, been investigated in detail so far.

Head movement is known to be a significant confounding factor in resting-state analysis of fMRI data (Murphy et al., 2013). It has been widely reported that head movement is a problem even when standard fMRI head-movement correction techniques are used (Yan et al., 2013, Mowinckel et al., 2012, Power et al., 2012, Van Dijk et al., 2012). These studies reveal that head-movement correction techniques can leave subtle head-movement effects behind, which can be mistaken for brain activity. Also, different levels of head movement during fMRI scans can result in differences in the allocation of network nodes in graph-theoretical analyses. Van Dijk and colleagues (Van Dijk et al., 2012) reported that the results of functional connectivity studies are altered when motion is regressed out from the estimate of functional connectivity. Power and colleagues (Power et al., 2012) proposed a “scrubbing” procedure to remove such left-over effects and reported that children's functional networks end up looking more like adult networks when that procedure is implemented. Van Dijk and colleagues (Van Dijk et al., 2012) also reported that movement during fMRI scans behaves like a personal trait and can affect participant-level results in ways that can yield systematic differences between populations that are unrelated to neural activity. Satterthwaite and colleagues (Satterthwaite et al., 2012) examined the effects of head movement on fMRI resting-state networks of children and young adolescents. They reported that head movement is strongly correlated with age and influences the functional connectivity metrics examined. Finally, Fair and colleagues (Fair et al., 2013) noted the need for stringent head movement correction strategies in resting-state analysis in their cohort of children and adolescents with Attention Deficit and Hyperactivity Disorder.

Given that head movement affects resting-state analyses in the case of fMRI, where motion correction techniques are routinely applied, it is important to understand whether head movement in the MEG system can affect MEG resting-state analyses and give inaccurate results. Despite the fact that the small size of the MEG helmet naturally applies restrictions to the amount participants can move their heads during the recording, head movements in the MEG system can vary significantly between participants. Some participants may be very still for most of the recording but move suddenly and end up at a very different position compared to the start of the recording. Other participants may continuously move by small distances around their starting head position. Yet other participants may slowly and progressively drift away from their position at the start of the recording. These qualitatively different movements can have very different effects on the results. It should also be added that participants with smaller heads (for example children) and participants from certain patient populations (for example people with Parkinson's disease) can exhibit a larger range of head movement than participants with larger heads or than healthy adults. It has recently been suggested that 3D-printed head casts can be effectively used to minimize head movement in the MEG system (Troebinger et al., 2014). Those head casts, however, need to be custom-made for each participant and therefore add to the monetary and time cost of MEG studies. For challenging groups, such as children and patients, these head casts may also not be well tolerated by all participants.

In this work we present the first, to our knowledge, systematic study of potential correlations between head movement in the MEG system and a) the beamformer envelope time series and b) functional networks, for MEG resting-state recordings. A lack of such correlations would give credibility to the results of the large number of existing MEG studies that use beamformer or network analyses to study source-localized function of the human brain at a systems level, in health and disease (for example Koelewijn et al., 2015, Demuru et al., 2014, Brookes et al., 2011, Stam et al., 2006, Stam et al., 2009; to list a few). We also explore and compare two software methods that aim to reduce the effects of any observed correlations from the analysis, thus increasing the power of future studies and rendering them more reliable.

## 2 Methods

### 2.1 Participants

Seventeen healthy participants (8 female) were recruited among the students and researchers of the School of Psychology at Cardiff University to participate in the study and volunteered without receiving compensation for their participation. Data from another 13 healthy participants (4 female) that had been previously acquired were also included in the study. Those 13 participants had been recruited from the local population of the Cardiff area to act as control participants in a different study and received payment for their participation. This resulted in 30 datasets available for analysis. All participants gave written informed consent. All procedures were approved by the local Ethics Committee.

### 2.2 Data acquisition

Data acquisition was performed in accordance with standard procedures for acquisition of MEG resting-state data at Cardiff University Brain Research Imaging Center (CUBRIC), (described in Koelewijn et al., 2015, Muthukumaraswamy et al., 2013). For completeness we provide the details here.

Whole-head MEG recordings were made using a 275-channel CTF radial gradiometer system and were sampled at 1200 Hz. An additional 29 reference channels were recorded for noise cancellation purposes and the primary sensors were analysed as synthetic third-order gradiometers (Vrba and Robinson, 2001). Three of the 275 channels were turned off due to excessive sensor noise. All participants also had a 3D structural magnetic resonance imaging (MRI) scan (1 mm isotropic voxel resolution, Fast Spoiled Gradient-Recalled-Echo pulse sequence), which was used for MEG source localization. To achieve MRI-MEG coregistration, 3 fiducial markers were placed at fixed distances from the nasion, the left pre-auricular point and the right pre-auricular point. These points were identifiable in each participant's anatomical MRI scan. The exact position of the 3 fiducial markers (coils) was verified during the analysis using high-resolution digital photographs that had been taken just before the MEG recording. Five electrodes were also placed above and below the center of the left eye, under the left and the right temple and behind the left ear, in order to record blinks and eye movements.

During the resting-state recordings, which lasted for 10 min, the participants were seated comfortably on the seat of the MEG system, in front of a grey screen. They rested their chin on an adjustable plastic chin-rest in order to minimize head movements. They were instructed to keep their eyes open and fixate on a red dot at the center of the screen for the duration of the recording, in order to minimize eye movements. Continuous head localization was achieved by recording the position of the 3 fiducial coils throughout the MEG recording, relative to the dewar of the MEG system. The resting-state recording was followed by two sessions of task-related recordings (not relevant to this study), each lasting approximately 17 min. Importantly, the resting-state recording always preceded the task-related recordings, in order to avoid the participants' resting-state brain activity being influenced by any remnant task-related effects.

### 2.3 Data analysis

Data processing was performed using MATLAB (MATLAB and Statistics Toolbox Release 2012b, The MathWorks, Inc., Massachusetts, Unites States).

MRI-MEG co-registration for all participants was performed based on the pictures taken, which showed the positions on which the 3 fiducial coils had been placed. The MEG data was down-sampled from 1200 Hz to 600 Hz by removing every other time point, divided into 2-s segments and visually inspected to identify possible artifacts. Artifacts were thought to be due to dental work, blinking and external factors. Segments on which such artifacts were present were excluded from the analysis. Data from participants that had fewer than 100 2-s segments remaining after artifact rejection were excluded from the analysis, so only participants for whom there was at least 200 s of good quality data were included. This requirement resulted in exclusion of 6 datasets, leaving datasets from 24 participants (10 female) appropriate for analysis. The age range of the participants used in the analysis was 18–55 years of age (mean: 31 years, standard deviation (SD): 10 years). The duration of the datasets used in the analysis was 206–568 s (mean: 438 s, SD: 93 s).

Beamformer calculation and network construction was performed as described by Brookes and colleagues (Brookes et al., 2011). Specifically, the data was first filtered into 6 frequency bands: delta (1–4 Hz), theta (4–8 Hz), alpha (8–13 Hz), beta (13–30 Hz), lower gamma (30–50 Hz) and upper gamma (50–90 Hz) using a least-squares finite impulse response band-pass filter. A Synthetic Aperture Magnetometry (SAM) beamformer algorithm was used to project the MEG data to source space and identify signal sources in the brain (Robinson and Vrba, 1998). Using the preprocessed data, beamformer weights were computed on an 8-mm grid for each participant and each frequency band. A multiple local-spheres volume conductor model (Huang et al., 1999) was derived by fitting spheres to the brain surface extracted by the FSL Brain Extraction Tool (Smith, 2002). Beamformer envelope time courses were then generated at every such grid point. In this paper, we refer to these grid points as ‘voxels’, because we follow the custom in the MEG literature to display source-space results as 3-dimensional volumetric images akin to the fMRI literature, so that we can best present the results using cut-through slices at each dimension. This has the added advantage of being more comparable to images derived using fMRI. It is important, however, to keep in mind that these ‘voxels’ reflect beamformer solutions for a cloud of points distributed in a grid. The voxel-based beamformer envelope time courses were subsequently normalized by an estimate of the projected noise amplitude at each voxel (Hall et al., 2013). For each of these beamformer time series, the amplitude envelope, calculated as the absolute value of the analytic signal (MATLAB function hilbert) was computed, temporally down-sampled to 1 Hz, transformed to the MNI (T1) average brain using FSL-FLIRT and concatenated across participants for each frequency band. High temporal correlation between envelopes was taken to imply connectivity of the associated brain areas, and thus network behavior.

Temporal independent component analysis (ICA) was applied to the concatenated data. For each frequency band, 25 independent components (ICs) were derived in order to identify networks of brain activity (Hyvarinen et al., 2010). To do this, pre-whitening was first applied to reduce the dimensionality of the source-space Hilbert envelope signals to 25 principal components (Hall et al., 2013, Brookes et al., 2011, Hyvarinen and Oja, 2000), followed by the fast-ICA algorithm (research.ics.tkk.fi/ica/fastica). After ICA computation, Pearson correlation between the temporal ICs and the time course of each voxel was calculated in order to measure the spatial signature of each temporal IC. Visual inspection of the resulting networks allowed identification of resting-state networks that have been reported previously in the literature (Brookes et al., 2011, Beckmann et al., 2005). Here we use the amplitude of temporal ICs resembling functional networks of interest to reflect network activation.

### 2.4 Head movement

Head movement was measured by the position of the three fiducial coils with respect to a coordinate system fixed on the dewar and was continuously obtained during the MEG recordings. The measures of head movement deemed relevant for this study were: a) the amount of motion of a fiducial coil within each 1-s interval (which is the time interval for which network activation is calculated) and b) the displacement of a fiducial coil with respect to its position at the beginning of the recording. To quantify the former aspect of movement, the amount of motion of each of the 3 fiducial coils between sample points of the recording was calculated for each second of the recording. In the following, we refer to that quantity as “instantaneous motion” for each fiducial coil. We note that the instantaneous motion can be regarded as a measure of the average velocity of that coil in each second of the recording (however because we do not divide by time, this metric has units of length). For the latter aspect of head movement, namely the displacement from the original position, we calculated the displacement of each of the 3 coils from their positions at the start of the recording for each sample point. In the following, we refer to that quantity as “displacement”, for each fiducial coil.

To further quantify the head movement for each participant individually, we performed a spectral analysis of the time series of each of the six head movement metrics for each participant. Specifically, the time series for each of the head movement metrics is expected to contain its power within a frequency range [0, f_top_]. We calculated the frequency f_top_ for all six head movement metric time series for each participant by using the MATLAB function obw, which gives the frequency band in which 99% of the power of a time series is found.

### 2.5 Correlations between head movement and beamformer envelope time series

To assess whether head movement correlates with the beamformer envelope time series in each voxel, that voxel's envelope was put into a linear regression analysis (function fitlm of MATLAB) that included all six head movement metrics as regressors (after those metrics had been z-transformed), separately for each of the 24 participants. Specifically, we assumed the following relation for the beamformer envelope time series with head movement, in each voxel of each participant:

(1) T = A + B_mN_ m_N_ + B_mL_ m_L_ + B_mR_ m_R_ + B_dN_ d_N_ + B_dL_ d_L_ + B_dR_ d_R_ + E

where T is the beamformer envelope time series, m_N/L/R_ is the instantaneous motion of the nasion, left and right fiducial coils respectively, d_N/L/R_ is the displacement of the nasion, left and right fiducial coils respectively, A is the intercept constant and E denotes the part of T that is unexplained by the model. The coefficients A and B were determined by the linear regression analysis.

We were specifically interested in how much variability in the beamformer envelope time series can be explained by the six head movement metrics in each voxel of each participant. Therefore, we report the coefficient of determination R^2^ for each such linear regression. For each participant, we calculated the percentage and the distribution of voxels for which the linear regression indicated a statistically significant relationship (*p* < 0.05). We also calculated the maximum (over all voxels of any one participant) amount of variability explained by the six head movement metrics. In order to control for possible false discoveries that result from the large number of voxels we are looking for correlations at, we applied a false discovery rate (FDR) controlling procedure for each participant (Benjamini and Yekutieli, 2001).

In order to eliminate the head movement effects from the beamformer envelope time series, we explored two possible head movement correction methods. As a first approach, we regressed out of the 1-Hz beamformer envelope time series the six head movement metrics, according to the relation

(2) T_clean_ = T − B_mN_ m_N_ − B_mL_ m_L_ − B_mR_ m_R_ − B_dN_ d_N_ − B_dL_ d_L_ − B_dR_ d_R_.

This is the same as the approach described by Stolk and colleagues (Stolk et al., 2013). We use the term ‘clean’ to describe the resulting time series, as is done in that paper.

As a second approach we applied a participant-specific high-pass filter on the beamformer envelope time series. As a high-pass frequency we used the frequency that resulted from the spectral analysis of the head movement metrics of each participant individually, as described above. The high-pass filter was applied to the beamformer envelope time series after that time series had been down-sampled to 1 Hz.

### 2.6 Correlations between head movement and network activity

To assess whether head movement correlates with the activation of resting-state networks, the component time course of each of the networks of interest was regressed over all six head movement metrics mentioned above (after those metrics had been z-transformed), for each of the 24 participants. Specifically, for each participant and each network activation time series we assumed the relation

(3) T^ntw^ = A^ntw^ + B^ntw^_mN_ m_N_ + B^ntw^_mL_ m_L_ + B^ntw^_mR_ m_R_ + B^ntw^_dN_ d_N_ + B^ntw^_dL_ d_L_ + B^ntw^_dR_ d_R_ + E^ntw^

where we use the superscript “ntw” to indicate that the quantities refer to networks. This analysis gave a value for R^2^ for each participant and each network, and this value indicates the amount of variance in the network activation time series that is explained by the six head movement metrics. For each network, each regression slope B in equation (3) was put into a 1-sample *t*-test to test the null hypothesis that the mean regression slope of the population is zero, which would correspond to no statistically significant correlation between the network time course and the relevant head movement metric.

To assess and understand the effect that regressing the six head movement metrics out of the beamformer envelope time series has on the resting-state networks, we performed the ICA on the ‘clean’ beamformer envelope time series, reconstructed the networks and repeated the correlation analysis with the head movement metrics as described in the previous paragraph. Similarly, we performed ICA on the high-pass filtered beamformer envelope time series and reconstructed the networks.

### 2.7 Changes in network maps

In order to assess whether the head movement correction leads to changes in the connectivity patterns we compared the spatial maps of the networks before and after head movement correction on the beamformer envelope time series. We treated each network as a vector in the space of voxels, the coordinates of each vector being the weights of the ICA un-mixing matrix which represent the contribution of each voxel to the map of the network (and therefore also reflect the connectivity among voxels within each network). In order to assess whether there are changes in the connectivity before and after head movement correction, we compared the vector of the weights of the un-mixing matrix for the networks generated with no head movement correction to those generated after head movement correction. We have 3386 such weights for each network, equal to the number of voxels that contribute to the networks we are looking into. We then used the *cosine similarity*, a measure of similarity between vectors, and the angle that results from that cosine. The cosine similarity for two vectors **V** = (V_1_, V_2_, V_3_, …, V_N_) and **W** = (W_1_, W_2_, W_3_, …, W_N_) is defined as

$$\cos\left(\theta \right)=\;\frac{\sum_{i=1}^{N}V_{i}W_{i}}{\sqrt{\sum_{i=1}^{N}V_{i}}\sqrt{\sum_{i=1}^{N}W_{i}}}$$

and the angle between the two vectors can be calculated by taking the inverse cosine of the result. A low value for the angle would indicate that the vectors are aligned and that the connectivity does not change due to head movement correction. A large value for the angle, on the other hand, would indicate significant differences in the connectivity before and after head movement correction.

## 3 Results

### 3.1 Head movement in the MEG system

In order to understand how and by how much different participants move in the MEG system, we examined how the head movement metrics defined in Sec. 2.4 vary among participants.

**Fig. 1:**
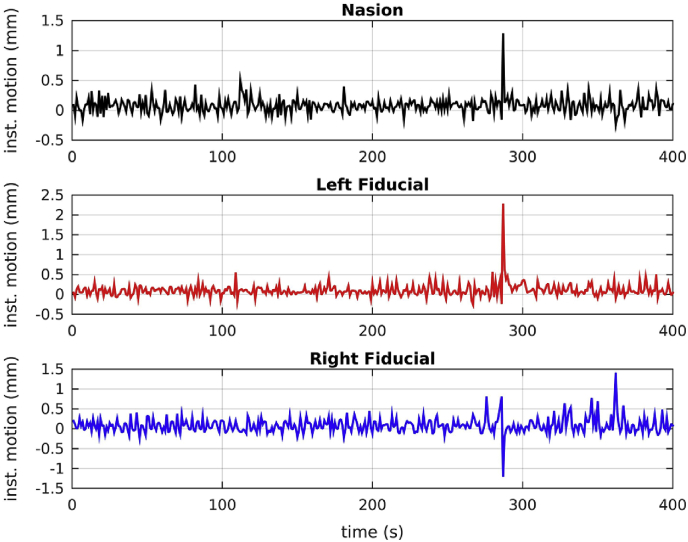
Instantaneous motion for the 3 fiducial coils for the first 400s of the recording of one participant. For most of the time the values of instantaneous motion for the 3 fiducial coils are small. There are, however, a few spikes indicating “jerky” movement.

The instantaneous motion for the three fiducial coils of one participant for the first 400 s of the recording is shown in Fig. 1. The participant shows relatively small instantaneous motion for most of the time, but also exhibits a few spikes of large value, indicating “jerky” movement. This is typical of all participants, although some participants show more spikes than others, or exhibit different upper and lower values of instantaneous motion.

**Fig. 2:**
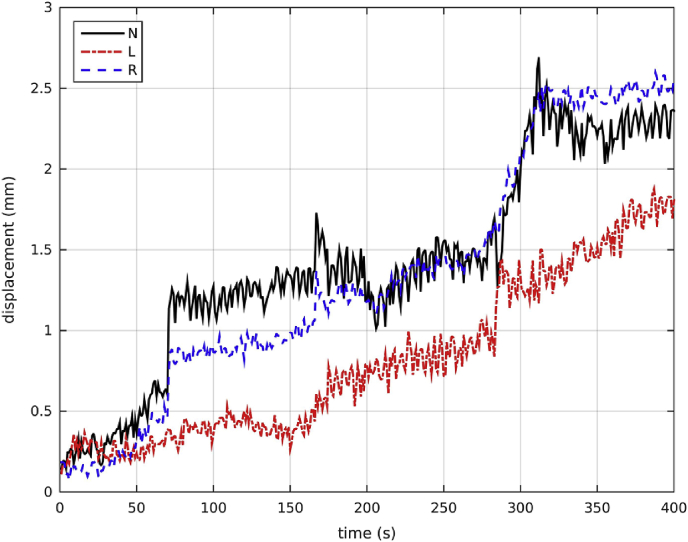
Displacement for the 3 fiducial coils for the first 400s of the recording of one participant. The displacement generally increases with time for all 3 coils. The (black) solid line indicates the nasion, the (red) dashed-dotted line indicates the left fiducial coil and the (blue) dashed line indicates the right fiducial coil.

The displacement from the starting position for one participant is shown in Fig. 2. The participant drifts away from the original position as the recording progresses. This drift, observed in the displacement time series of all participants, is in the downward direction, and suggests that as the recording progresses participants tend to relax more into the seat of the MEG system. This is in agreement with the downward drift of young participants reported by Wehner et al. (2008).

The mean and SD for the six head movement metrics are given in Table 1 for all participants. The range of values for the instantaneous motion (0.023–0.166 mm) and the displacement (0.405–7.771 mm) show that our study includes participants exhibiting very different ranges of head movement. The group means and standard deviations for those quantities are given in Table 2.

**Table 1:** Mean and SD (over the duration of the recording) of the six head movement metrics for the 24 participants. (N = nasion, L/R = left/right pre-auricular point).

Participant Num.	Inst Motion N (mm)		Inst Motion L (mm)		Inst Motion R (mm)		Displacement N (mm)		Displacement L (mm)		Displacement R (mm)	
	Mean	SD	Mean	SD	Mean	SD	Mean	SD	Mean	SD	Mean	SD
1	0.027	0.028	0.023	0.030	0.049	0.049	1.080	0.267	1.025	0.413	0.405	0.158
2	0.030	0.033	0.030	0.030	0.051	0.049	2.165	1.107	0.821	0.427	1.001	0.563
3	0.033	0.037	0.030	0.047	0.061	0.086	1.508	0.671	0.922	0.577	1.462	0.812
4	0.032	0.031	0.033	0.061	0.054	0.060	0.813	0.443	1.658	0.877	1.804	0.856
5	0.081	0.125	0.102	0.163	0.082	0.188	3.670	2.388	2.677	1.755	3.341	2.166
6	0.036	0.041	0.036	0.039	0.054	0.052	1.424	0.569	1.445	0.651	0.956	0.392
7	0.033	0.033	0.029	0.035	0.052	0.057	1.190	0.273	1.359	0.366	2.221	0.506
8	0.042	0.069	0.032	0.047	0.068	0.112	2.851	1.889	1.062	0.356	1.114	0.683
9	0.035	0.041	0.030	0.034	0.047	0.044	0.463	0.121	0.900	0.487	1.012	0.526
10	0.043	0.051	0.040	0.046	0.058	0.079	1.356	0.521	1.083	0.458	0.769	0.681
11	0.036	0.033	0.034	0.050	0.061	0.060	1.607	0.446	1.321	0.376	1.791	0.666
12	0.043	0.047	0.038	0.093	0.105	0.086	7.235	3.114	7.771	3.412	4.221	1.953
13	0.068	0.141	0.067	0.110	0.166	0.249	4.472	1.701	3.857	1.394	6.392	2.995
14	0.057	0.057	0.046	0.063	0.078	0.091	1.683	0.427	2.105	0.434	1.180	0.343
15	0.045	0.054	0.033	0.094	0.065	0.067	1.667	1.038	2.136	1.174	2.177	0.754
16	0.12	0.112	0.115	0.119	0.166	0.176	0.718	0.232	1.203	0.345	1.035	0.428
17	0.049	0.189	0.049	0.121	0.079	0.094	7.312	3.307	5.786	2.735	5.938	3.114
18	0.028	0.039	0.030	0.0476	0.058	0.059	0.929	0.475	1.671	0.930	2.328	1.103
19	0.047	0.047	0.039	0.057	0.065	0.063	0.910	0.510	0.955	0.454	0.733	0.337
20	0.054	0.093	0.050	0.137	0.144	0.200	1.512	0.481	1.051	0.318	0.892	0.321
21	0.036	0.042	0.043	0.052	0.060	0.058	0.452	0.134	0.981	0.484	0.491	0.166
22	0.042	0.065	0.053	0.120	0.053	0.098	1.891	1.314	1.929	1.073	2.242	1.122
23	0.041	0.040	0.028	0.067	0.049	0.054	2.977	1.708	2.472	1.623	1.896	0.900
24	0.062	0.060	0.069	0.082	0.103	0.234	2.499	1.132	1.366	0.610	2.257	0.875

**Table 2:** Mean and SD (over the 24 participants) for the six head movement metrics.

	Inst Motion Nasion (mm)	Inst Motion Left Fid (mm)	Inst Motion Right Fid (mm)	Displ Nasion (mm)	Displ Left Fid (mm)	Displ Right Fid (mm)
Mean	0.047	0.045	0.076	2.168	1.981	1.986
SD	0.020	0.023	0.035	1.860	1.664	1.572

**Fig. 3:**
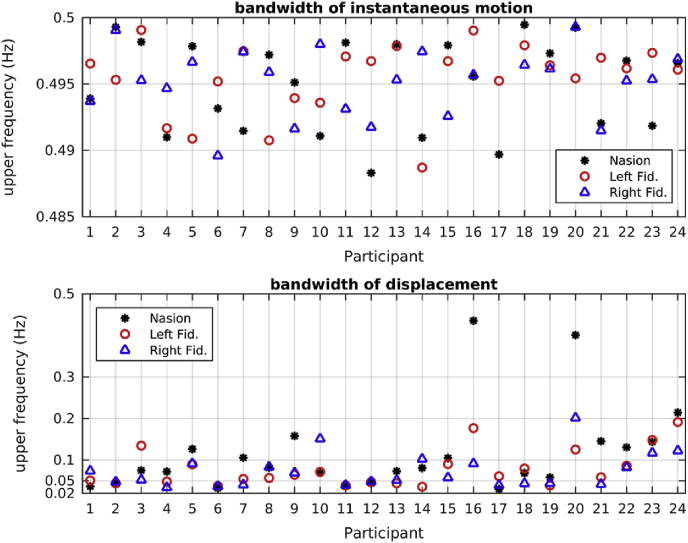
Top frequencies for the instantaneous motion (top panel) and for the displacement bottom panel of the 3 fiducial coils for the 24 participants. The black dots indicate the nasion, the red circles indicate the left fiducial coil and the blue triangles indicate the right fiducial coil. In order to show the frequencies for each participant clearly for the case of the displacement, we are using a logarithmic scale on the y-axis of that plot.

Fig. 3 shows the ‘top’ frequency f_top_ that is the upper limit of the frequency band where the power of the time series for the instantaneous motion and for the displacement is concentrated, for each coil and each participant. As expected, both time series have their power concentrated in low frequencies, with the instantaneous motion exhibiting higher top frequencies.

As mentioned earlier, we explored applying a high-pass filter to the beamformer envelope time series to correct for head movement effects. As can be seen in Fig. 3, for each participant there are six different frequencies that can be used as cutoff frequencies in the high-pass filter. For reasons that we will detail in Sec. 3.3, we choose as the high-pass filter cutoff frequency for each participant the highest of the 3 frequencies resulting from the spectral analysis of the *displacement* of the 3 fiducial coils for that participant.

### 3.2 Correlations between head movement and amplitude envelope beamformer time series

For each frequency band, we performed a linear regression of the beamformer envelope time series for each voxel of each participant jointly over all six head movement metrics. The linear regression gave the coefficient of determination R^2^, a measure of the total variability in the amplitude envelope that can be explained by all six head movement metrics together, and a corresponding *p*-value.

To quantify the correlations, we calculated the maximum (over all voxels) R^2^ for each participant separately for each frequency band (with statistical significance at *p* < 0.05), which is the maximum percentage variance of the beamformer envelope time series explained by the head movement metrics. We also calculated, for each participant, the percentage of voxels exhibiting non-zero R^2^ for correlations that were statistically significant at the *p* < 0.05 level. Given that each participant has of the order of 6000 voxels, we use a false detection rate (FDR) procedure to control for multiple comparisons for each participant.

**Fig. 4:**
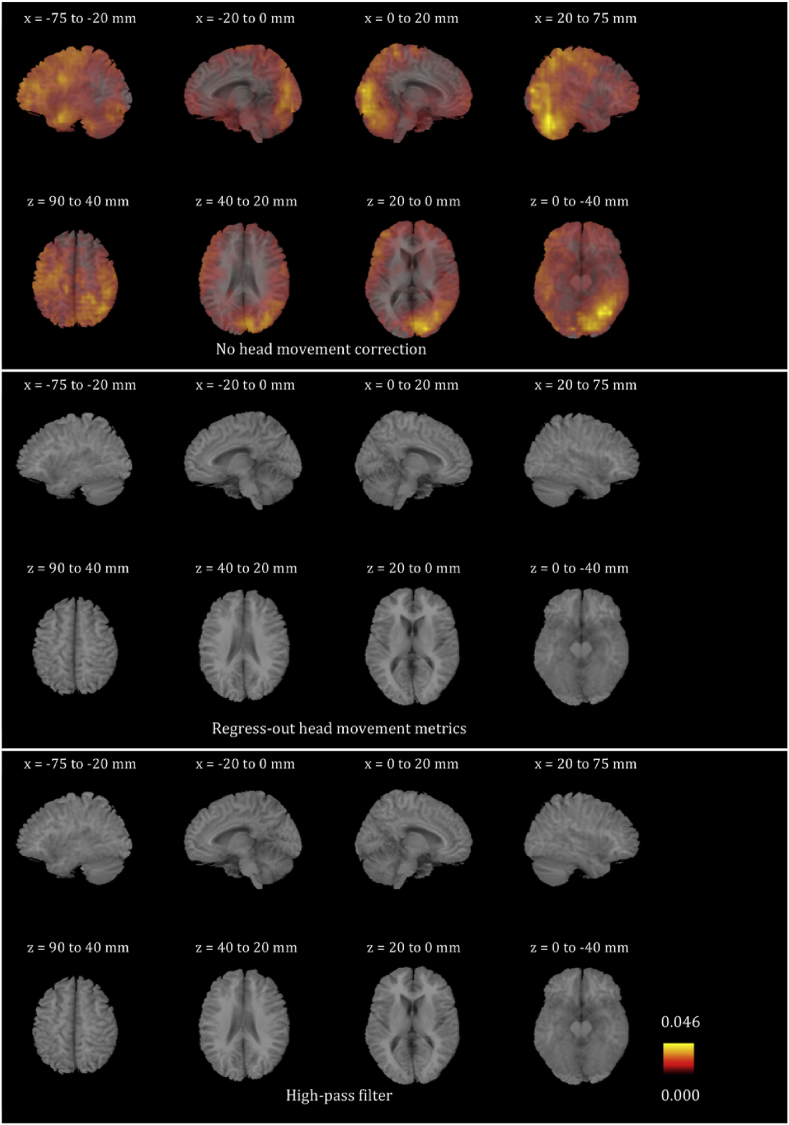
Mean (over the 24 participants) maps of the R^2^ of the voxel-wise correlations between the beamformer envelope time series and the head movement metrics, for the delta frequency band. The three panels, from top to bottom, show the maps for no head movement correction, for head movement correction resulting by regressing the head movement metrics out of the beamformer envelope time series, and for head movement correction resulting from the participant-specific high-pass filter. No correlations remain in the group after either of the two head movement correction methods is applied.

**Fig. 5:**
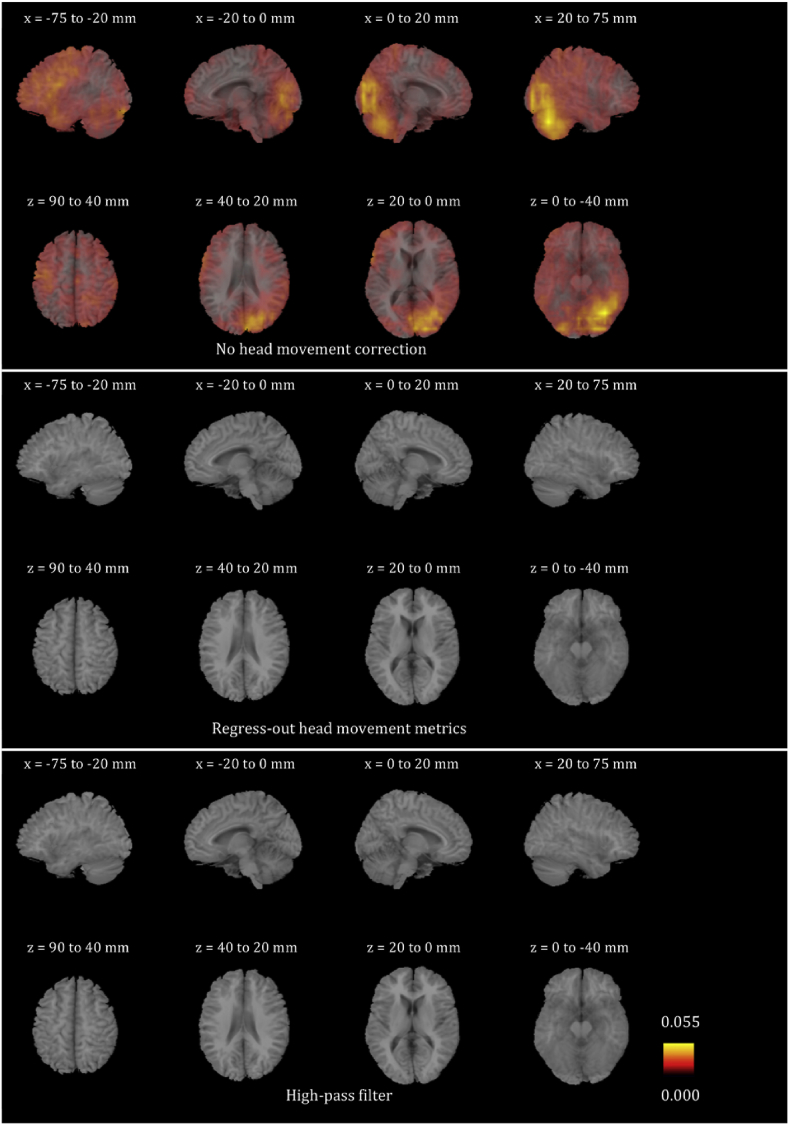
Mean (over the 24 participants) maps of the R^2^ of the voxel-wise correlations between the beamformer envelope time series and the head movement metrics, for the theta frequency band. The three panels, from top to bottom, show the maps for no head movement correction, for head movement correction resulting by regressing the head movement metrics out of the beamformer envelope time series, and for head movement correction resulting from the participant-specific high-pass filter. No correlations remain in the group after either of the two head movement correction methods is applied.

**Fig. 6:**
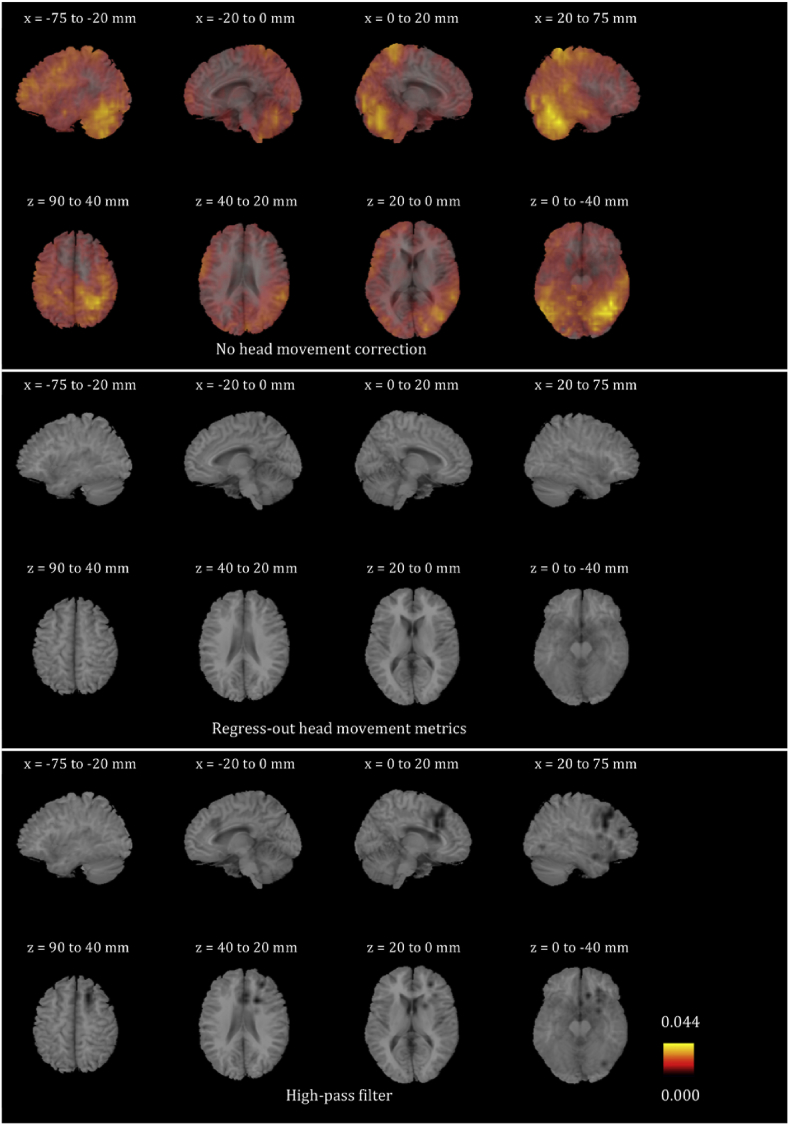
Mean (over the 24 participants) maps of the R^2^ of the voxel-wise correlations between the beamformer envelope time series and the head movement metrics, for the alpha frequency band. The three panels, from top to bottom, show the maps for no head movement correction, for head movement correction resulting by regressing the head movement metrics out of the beamformer envelope time series, and for head movement correction resulting from the participant-specific high-pass filter. No correlations remain in the group after regressing out the head movement metrics, while some small values of R^2^ remain after the participant-specific high-pass filter.

**Fig. 7:**
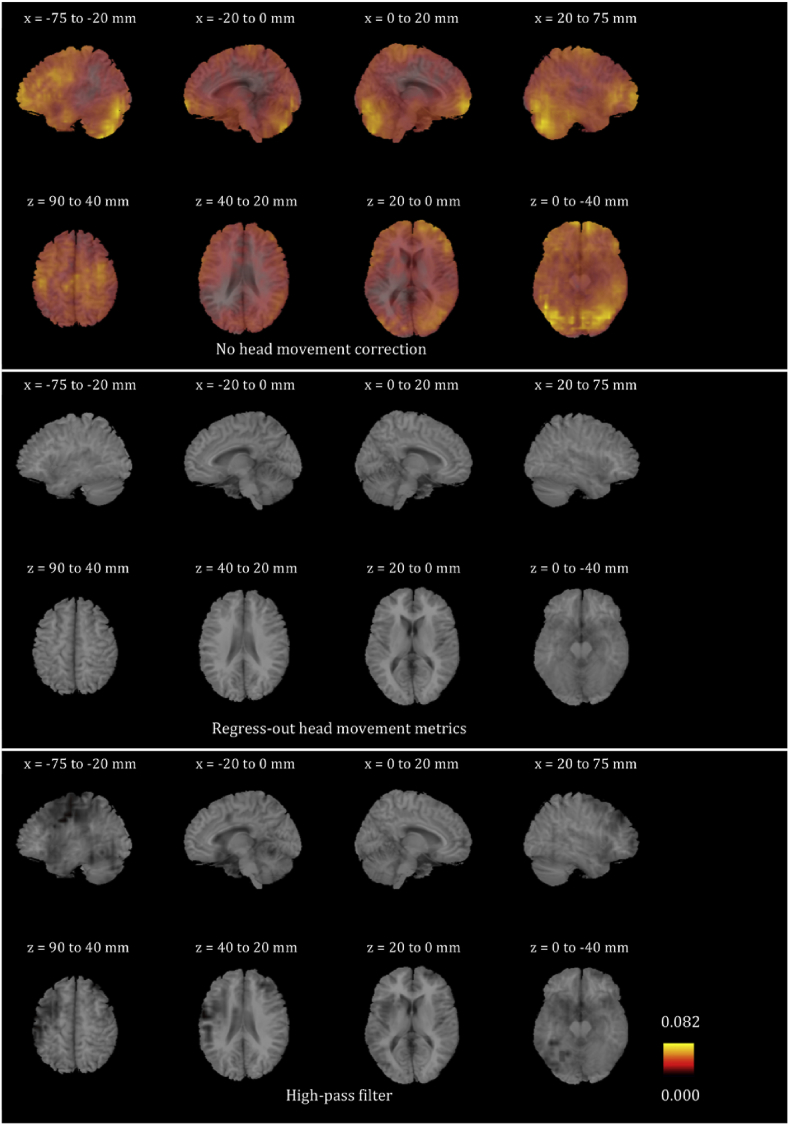
Mean (over the 24 participants) maps of the R^2^ of the voxel-wise correlations between the beamformer envelope time series and the head movement metrics, for the beta frequency band. The three panels, from top to bottom, show the maps for no head movement correction, for head movement correction resulting by regressing the head movement metrics out of the beamformer envelope time series, and for head movement correction resulting from the participant-specific high-pass filter. No correlations remain in the group after regressing out the head movement metrics, while some small values of R^2^ remain after the participant-specific high-pass filter.

**Fig. 8:**
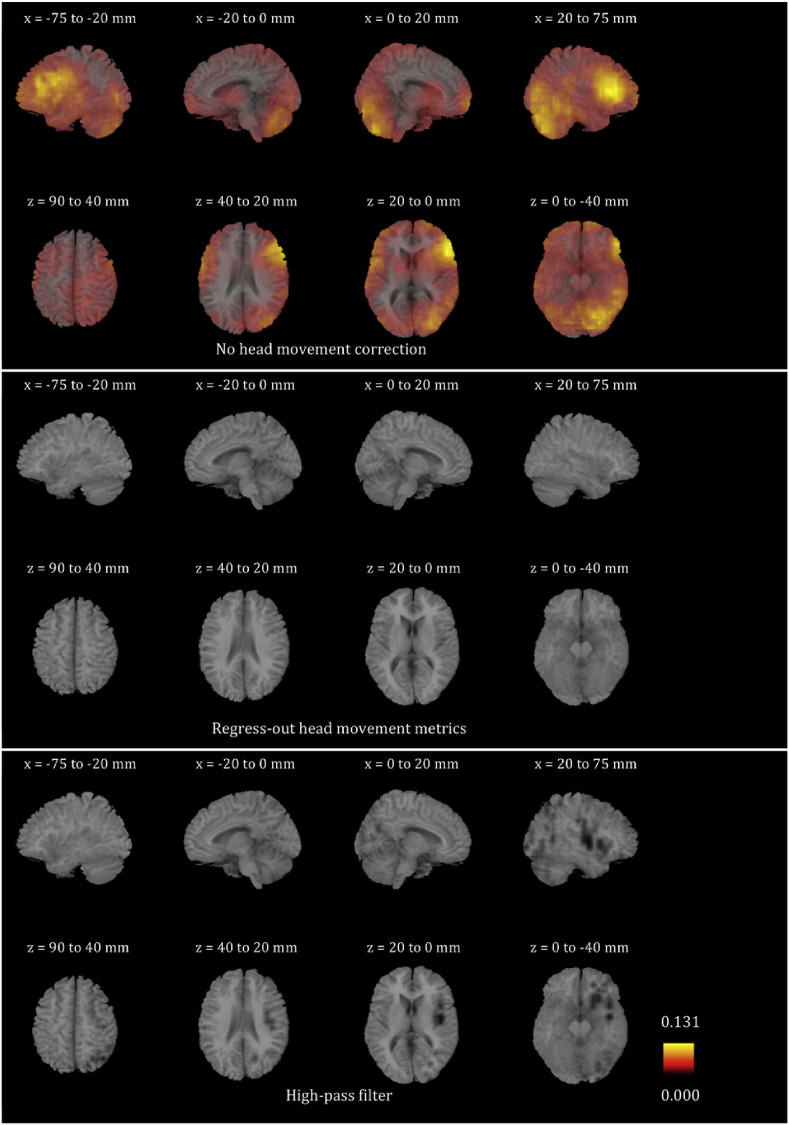
Mean (over the 24 participants) maps of the R^2^ of the voxel-wise correlations between the beamformer envelope time series and the head movement metrics, for the lower gamma frequency band. The three panels, from top to bottom, show the maps for no head movement correction, for head movement correction resulting by regressing the head movement metrics out of the beamformer envelope time series, and for head movement correction resulting from the participant-specific high-pass filter. No correlations remain in the group after regressing out the head movement metrics, while some small values of R^2^ remain after the participant-specific high-pass filter.

**Fig. 9:**
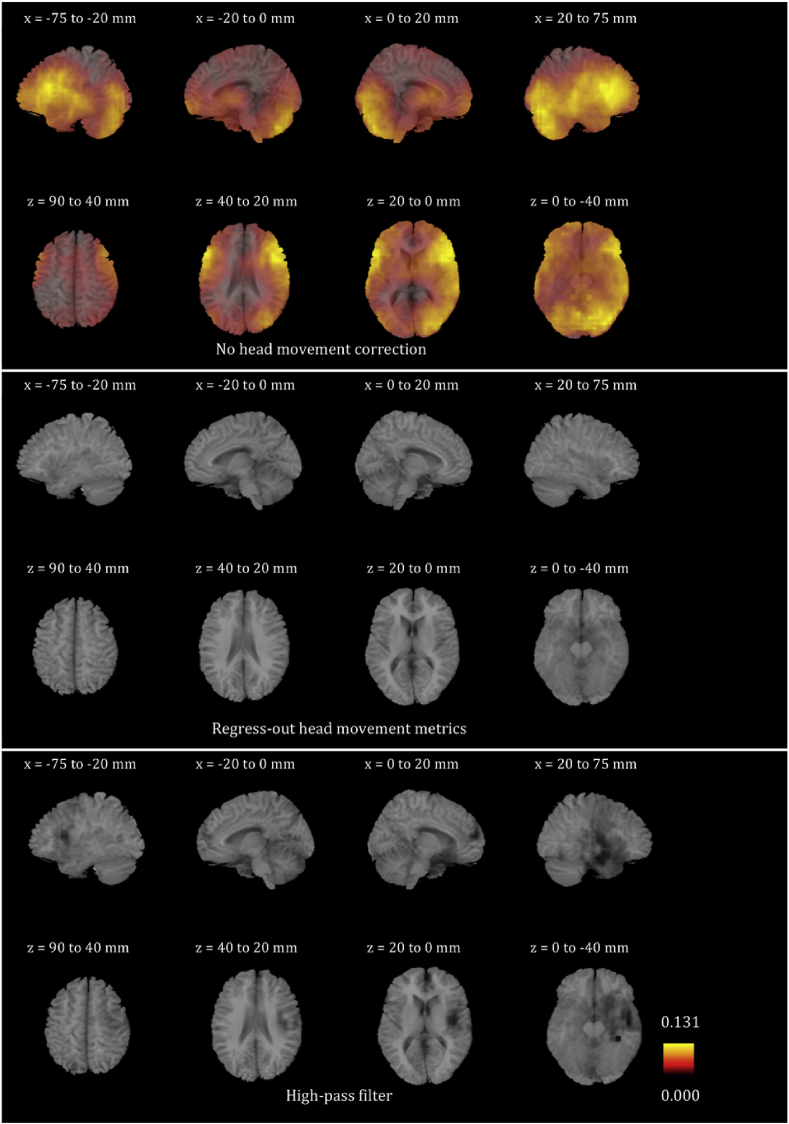
Mean (over the 24 participants) maps of the R^2^ of the voxel-wise correlations between the beamformer envelope time series and the head movement metrics, for the upper gamma frequency band. The three panels, from top to bottom, show the maps for no head movement correction, for head movement correction resulting by regressing the head movement metrics out of the beamformer envelope time series, and for head movement correction resulting from the participant-specific high-pass filter. No correlations remain in the group after regressing out the head movement metrics, while some small values of R^2^ remain after the participant-specific high-pass filter.

We plotted the voxel-wise map of R^2^ for all participants for the six frequency bands. These maps look very different for different participants. In order to give the reader a sense of the differences, we present in Appendix A the voxel-wise maps for three participants for all frequency bands. We only show the maps before any head movement correction has been applied to the beamformer envelope time series, because, as will become evident below, the maps after head movement correction are essentially blank due to the fact that head movement effects disappear. We also averaged the images for all 24 participants for each of the six frequency bands, and are showing the results in Fig. 4, Fig. 5, Fig. 6, Fig. 7, Fig. 8, Fig. 9. Based on these images, we can identify some trends regarding the areas that are most affected by head movement. Specifically, for the lower frequency bands (delta, theta, alpha and beta), it is the posterior areas that are affected more severely by head movement. In the case of the lower gamma band, the frontal areas that are mostly affected. In the case of the upper gamma band, both frontal and occipital areas are affected by head movement.

**Fig. 10:**
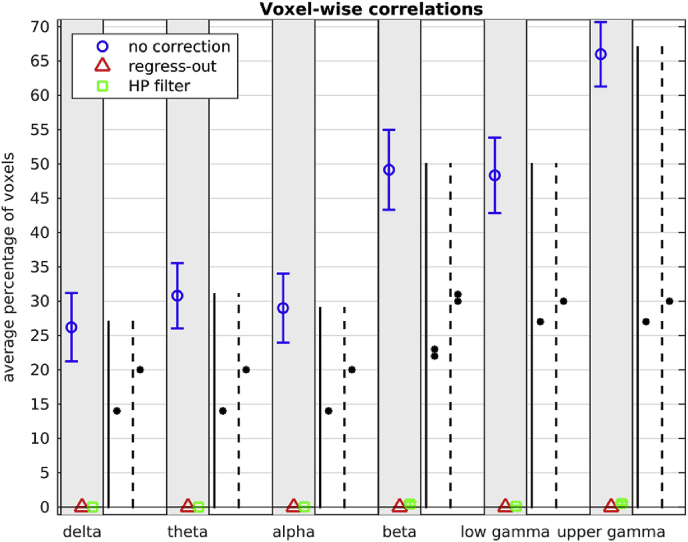
Average (over participants) percentage of voxels with non-zero coefficient of determination R^2^ (FDR correction applied for each participant), resulting from linear regression of the beamformer envelope time series with the 6 head movement metrics, in all frequency bands. The blue circles are for correlations without head movement correction applied, the red triangles are for head movement correction resulting from regressing the head movement metrics out of the beamformer envelope time series, and the green squares are for head movement correction resulting from high-pass filtering the beamformer envelope time series. The error bars show the standard error over the 24 participants. The stars indicate the significance for the paired *t*-test between the mean value without head movement correction and that resulting from head movement resulting from regressing-out the head movement metrics (solid line), between the mean value without head movement correction and that resulting from the participant-specific high pass filter (dashed line): * indicates *p* < 10^−4^, ** indicates *p* < 10^−10^.

**Fig. 11:**
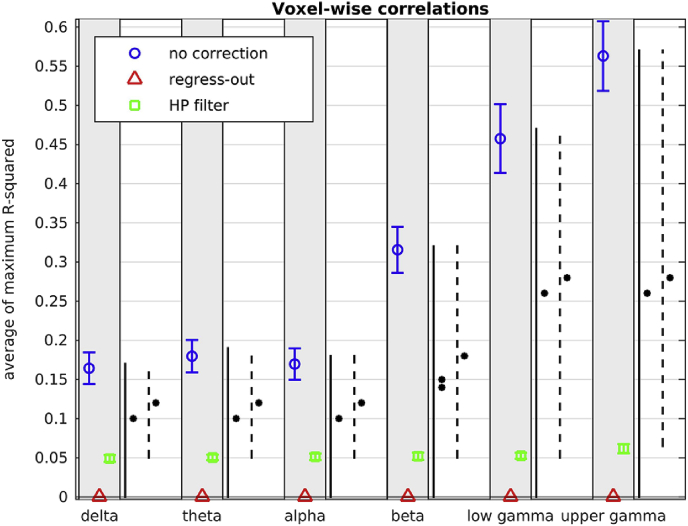
Average (over participants) of the maximum (over all voxels of each participant) coefficient of determination R^2^, resulting from linear regression of the beamformer envelope time series with the six head movement metrics, in the six frequency bands. The blue circles are for correlations without any head movement correction applied, the red triangles are for correction resulting from regressing the head movement metrics out of the beamformer time series, and the green squares are for correction resulting from the participant-specific high-pass filter of the beamformer envelope time series. The error bars show the standard error over the 24 participants. The stars indicate the significance for the paired *t*-test between the mean value without head movement correction and that resulting from head movement resulting from regressing-out the head movement metrics (solid line), between the mean value without head movement correction and that resulting from the participant-specific high pass filter (dashed line): * indicates *p* < 10^−4^, ** indicates *p* < 10^−10^.

The average (over participants) percentage of voxels with non-zero R^2^ and the average (over participants) of the maximum R^2^ is shown in Fig. 10, Fig. 11 respectively, for no head movement correction, for head movement correction resulting from regressing the six head movement metrics out of the beamformer envelope time series, and for head movement correction resulting from the participant-specific high-pass filter on the beamformer envelope time series. Fig. 10 shows that, if no head movement correction is applied, the beamformer envelope time series is plagued by statistically significant correlations with head movement for a large percentage of voxels. This percentage is higher for the upper frequency bands, reaching up to 70% for the upper gamma band. Fig. 11 shows that, if no head movement correction is applied, there are voxels for which a large proportion of the variability observed in the beamformer envelope time series can be explained by head movement, with R^2^ reaching up to 0.6 for some participants in the upper gamma frequency band.

If no head movement correction is applied, the average number of voxels affected by correlations with head movement is relatively constant across frequencies for the delta, theta and alpha bands, but increases with increasing frequency for the beta, lower and upper gamma bands (significance of *p* = 1.4 × 10^−2^, 2.1 × 10^−2^ and 6.9 × 10^−6^ for paired *t*-test between alpha-beta, alpha-lower gamma and alpha-upper gamma bands respectively). A similar trend is evident for the average value of the maximum R^2^ for no head movement correction (significance of *p* = 1.7 × 10^−4^, 3.2 × 10^−7^ and 2.4 × 10^−10^ for paired *t*-test between alpha-beta, alpha-lower gamma and alpha-upper gamma bands respectively). The fact that higher frequency beamformer envelope time series are affected more by head movement is possibly due to the fact that in resting-state recordings the lower-frequency time series have more neural-activity related content than the higher-frequency ones. For that reason, a larger proportion of the variability present in the higher frequency time series can be explained by head movement than is the case for lower frequencies.

It is important to understand whether all six head movement metrics affect the beamformer envelope time series to the same extent, when no head movement correction is applied. To assess that, we performed a 1-sample *t*-test on the linear regression slopes of all participants for each voxel for which the linear regression came out statistically significant (after multiple comparison correction), to test the null hypothesis that the mean regression slope of the population is zero, for that voxel. We report in Table 3 the number of voxels for which the null hypothesis of zero average slope is rejected, indicating that those voxels are affected by the respective head movement metric. In the beta frequency band, the beamformer envelope time series is affected by the nasion displacement for approximately a quarter of the voxels. In the upper gamma frequency band, the beamformer envelope time series is affected by the left fiducial displacement for approximately a quarter of the voxels and by the right fiducial displacement for approximately a fifth of the voxels.

**Table 3:** Number of voxels for which the mean (over participants) linear regression slope is statistically different from zero, for the six head movement metrics in each of the six frequency bands. N = nasion, L/R = left/right pre-auricular points.

	delta	theta	alpha	beta	lower gamma	upper gamma
Displ N	144	261	341	1506	653	40
Displ L	296	265	131	458	610	1686
Displ R	249	290	396	308	507	1312
Ins Mot N	358	182	213	574	596	283
Ins Mot L	249	192	285	105	159	673
Ins Mot R	130	186	176	442	192	127

The participant-specific high-pass filter of the beamformer envelope time series is very effective at reducing the percentage of voxels affected by head movement to 0.03%, 0.46%, 0.10% and 0.52% for the alpha, beta, lower gamma and upper gamma bands respectively and to 0 for the delta and theta bands. Regressing the head movement metrics out of the beamformer envelope time series is even more effective and reduces the percentage of voxels exhibiting statistically significant correlations to zero. The two head movement correction methods drastically reduce the maximum R^2^ as well. Regressing out the head movement metrics in particular reduces the values for the maximum R^2^ to nearly 0 for most participants.

To ascertain that the mean values shown in Fig. 10, Fig. 11 are statistically different before and after head movement corrections are applied, we perform a paired *t*-test on the means before head movement correction and after each of the head movement correction methods. All paired t-tests show that the means before and after head movement correction are different, with high significance (all *p* values are lower than 10^−4^).

### 3.3 Network identification

**Fig. 12:**
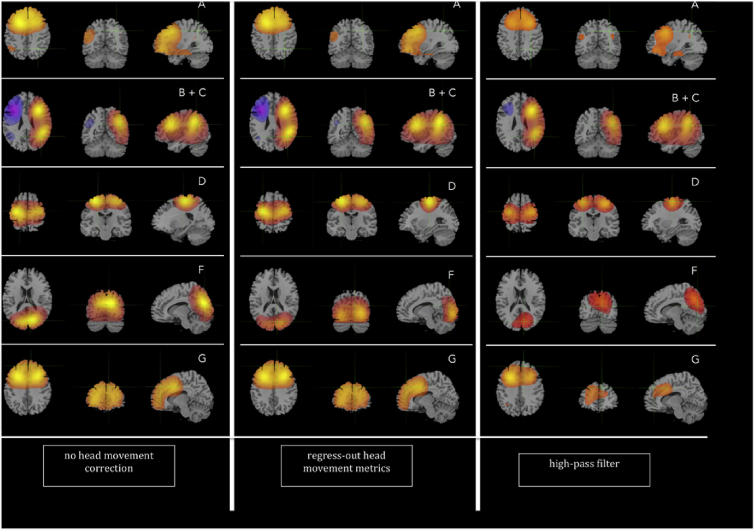
Networks derived in the present analysis (neurological convention). The 3 panels, from left to right, show the networks derived without any head movement correction (left), with head movement correction resulting from regressing the head movement metrics out of the beamformer envelope time series (center) and with head movement correction resulting from the participant-specific high-pass filter applied on the beamformer envelope time series (right). Each of the five rows shows, from left to right, the axial, coronal and sagittal views of each network. From top to bottom: A: default mode network (alpha band), B + C: left and right frontoparietal networks (beta band), D: sensorimotor network (beta band), F: visual network (beta band) and G: frontal lobes, including the anterior cingulate cortex (ACC) (beta band). The same scale has been used for each network before and after head movement correction, however the scales are not the same for different networks.

Visual inspection of the 25 ICs for each frequency band resulted in identification of the networks derived in Brookes et al. (2011). The networks are shown in the left panels of Fig. 12.

The default mode network (DMN, denoted by A in Brookes et al., 2011) was identified in the alpha frequency band. It appears to have more diffuse frontal activation than in Fig. 1 of Brookes et al., but is in good agreement with Fig. S7 of the supplementary material of the same paper. Brookes and colleagues identify the left-lateralized (denoted by B) and the right-lateralized (denoted by C) frontoparietal networks as two independent components in the beta frequency band, however in the present analysis the two networks were identified as one component, with the left- and right-lateralized parts exhibiting anti-correlated activity (second row of Fig. 1). This result has been obtained independently by other members of the CUBRIC group in some of the ICA analyses that involved different cohorts. To remain in notational agreement with Brookes et al., 2011 we denote this combined network as B + C. The sensorimotor network (D), visual network (F) and the frontal lobes (including the anterior cingulate cortex (ACC), denoted as G) were also identified in the beta band. None of the components that resulted from the ICA exhibited sufficient similarity with the medial parietal region network listed in Brookes et al. (2011) (denoted by E in their paper), so that component is not used in this study.

As a measure of activation within each of these networks, we use the relevant network's independent component time course.

The ICA was used three times, to produce three sets of networks. First it was used on the beamformer envelope time series without any correction for head movement, resulting in the networks shown in the left panel of Fig. 12. Second, it was used on the beamformer envelope time series after the six head movement metrics had been regressed out. This resulted in the networks shown in the central panel of Fig. 12. Finally it was performed on the beamformer envelope time series that had been high-pass filtered with the participant-specific high-pass filter described above. This resulted in the networks shown in the right panel of Fig. 12. When using the ICA for this latter case, we had to make a choice of which frequency we would use as the cutoff frequency in the high-pass filter. Ideally we would like to use the highest of the six frequencies identified for each participant and shown in Fig. 3, so that as much of the head movement contributions to the signal are excluded as possible. This meant using the highest of the instantaneous motion top frequencies (which is close to our Nyquist frequency of 0.5 Hz). Using that as the cutoff frequency of the high-pass filter resulted in no networks being reproduced at all. For that reason, when high-pass filtering the beamformer envelope time series, we used the largest of the 3 frequencies resulting from the spectral analysis of the 3 displacement time series for each participant. Using that cutoff frequency in our high-pass filter and repeating the ICA resulted in the networks shown in the right panel of Fig. 12. Given the cutoff frequency we use in the high-pass filter, we do not expect that all head movement effects will be eliminated from the resting-state measures we are investigating. Ultimately the performance of the high-pass filter depends on the percentage of the head movement effects due to instantaneous motion that is within the frequency range of the displacement.

The networks were reproduced in all three cases, but at first glance it is clear that some differences exist in the resulting maps for certain networks. In the following we look into whether the head movement correction leads to a change in the network activation time series and/or a change in the spatial connectivity pattern of the networks. Because the participant-specific high-pass filter was not as successful as regressing-out the head movement metrics in eliminating the head movement effects from the beamformer envelope time-series, we have shown the resulting networks in Fig. 12 for completeness but we do not look further into the correlations of those networks with head movement metrics.

### 3.4 Correlations between head movement and network activation

**Fig. 13:**
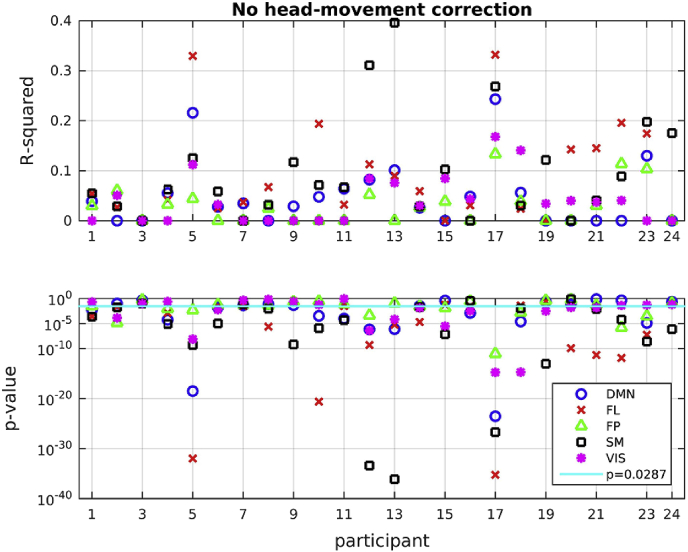
R-squared and *p*-values for all participants and all networks. The vertical axis scale of the *p*-value plot is logarithmic, and the *p*-values assume very low values, some as low as 10^−40^, indicating that the relationship is highly statistically significant. Networks: DMN = default-mode (circles), FL = frontal lobe (x), FP = frontoparietal (triangles), SM = sensorimotor (squares), VIS = visual (stars). The horizontal line in the plot with the *p*-values shows *p* = 0.0287, which is the threshold resulting from the FDR correction for multiple comparisons.

The time course for each of the 5 networks was put into a linear regression analysis over the six (z-transformed) head movement metrics for each of the 24 participants. We first report the value of R^2^ for each participant for each network, before any head movement correction has been applied. We only keep R^2^ if the linear regression has a *p*-value smaller than 0.05, i.e. it satisfies our significance criterion; when that criterion is not satisfied we set R^2^ = 0. The results are shown in Fig. 13. In the same figure we also show the related *p*-values. The values of R^2^ vary a lot for the different participants and networks, with several of the values reaching above 0.1, in some cases as high as 0.4, indicating that a large percentage of the variance observed in the network activation time series is explained by the head movement metrics. It is also noteworthy that the *p*-values are very low. Applying a FDR correction for multiple comparisons we get a threshold of 0.0287 for the comparisons we are performing; 69 out of the 120 comparisons survive that threshold, and 27 of those pertain to R^2^ values above 0.1. Importantly, it is clear that head movement affects the beamformer envelope time series differently for different participants. This last point is crucial because it implies that, if no head movement correction is applied in the activation time series, we are mixing, in our analyses, different amounts of physiological and non-physiological signals for different participants. Such an approach is certain to lead to erroneous results in cases where participant populations with different characteristics are compared with each other, for example patients versus healthy participants, or young versus old participants.

For each network, the linear regression slopes of the participants were put into a 1-sample *t*-test to test the null hypothesis that the mean regression slope of the population is zero, which would correspond to no statistically significant correlation between network activation and the head movement metric in question. This analysis was performed once for networks derived without head movement correction and once for networks derived after the six head movement metrics had been regressed out of the beamformer envelope time series. The results are shown in Table 4, Table 5 respectively.

**Table 4:** Slopes resulting from the linear regression of network activation with the six head movement metrics, when no head movement correction is applied. Bold italics indicate statistically significant relations at *p* < 0.05 level (uncorrected). N = nasion, L/R = left/right pre-auricular points. Networks: DMN = default-mode, FP = frontoparietal, SM = sensorimotor, VIS = visual, FL = frontal lobe.

		DMN	FP	SM	VIS	FL
Instantaneous Motion - Nasion	Mean slope	***0.022***	0.005	−0.027	0.004	**−*0.020***
	p-value	***0.023***	0.607	0.065	0.766	***0.041***
Instantaneous Motion – Left Fiducial	Mean slope	−0.011	0.001	−0.014	0.019	0.017
	p-value	0.318	0.914	0.211	0.147	0.130
Instantaneous Motion–Right Fiducial	Mean slope	0.013	−0.006	−0.010	−0.009	−0.013
	p-value	0.175	0.609	0.361	0.453	0.205
Displacement – Nasion	Mean slope	**−*0.236***	−0.109	0.268	0.058	***0.269***
	p-value	***0.009***	0.105	0.057	0.653	***0.030***
Displacement – Left Fiducial	Mean slope	0.091	−0.040	**−*0.478***	0.224	−0.084
	p-value	0.347	0.656	***0.004***	0.088	0.518
Displacement – Right Fiducial	Mean slope	***0.163***	0.112	0.022	**−*0.228***	−0.104
	p-value	***0.049***	0.199	0.863	***0.017***	0.385

**Table 5:** Slopes resulting from the linear regression of network activation with the six head movement metrics, when correcting for head movement by regressing out the six head movement metrics. For ease of presentation we have multiplied all values of the slopes by 10^10^. For example, the slope of the DMN with instantaneous motion of the nasion is −0.237 × 10^−10^. N = nasion, L/R = left/right pre-auricular points. Networks: DMN = default-mode, FP = frontoparietal, SM = sensorimotor, VIS = visual, FL = frontal lobe.

		DMN	FP	SM	VIS	FL
Instantaneous Motion - Nasion	Mean slope (×10^10^)	−0.237	−0.791	2.490	−2.546	−0.021
	p-value	0.784	0.649	0.354	0.246	0.987
Instantaneous Motion – Left Fiducial	Mean slope (×10^10^)	0.384	0.756	1.578	−0.672	1.313
	p-value	0.698	0.742	0.511	0.758	0.340
Instantaneous Motion–Right Fiducial	Mean slope (×10^10^)	0.089	−2.660	0.096	3.890	−0.584
	p-value	0.930	0.205	0.956	0.197	0.748
Displacement – Nasion	Mean slope (×10^10^)	6.253	−24.35	−25.22	2.196	0.907
	p-value	0.204	0.222	0.179	0.872	0.943
Displacement – Left Fiducial	Mean slope (×10^10^)	0.346	31.38	−5.672	−6.876	3.434
	p-value	0.934	0.103	0.734	0.578	0.824
Displacement – Right Fiducial	Mean slope (×10^10^)	−6.764	−6.037	29.27	5.722	−4.676
	p-value	0.195	0.588	0.168	0.646	0.635

If no head movement correction is applied, several statistically significant correlations exist between the network activation and the head movement metrics (Table 4). Specifically, the default mode network (DMN), the frontal lobe network (FL), the sensorimotor network (SM) and the visual network (VIS) all exhibit correlations with the displacement of some of the fiducial coils. The former two also exhibit correlations with the instantaneous motion of the nasion fiducial coil. More correlations are observed with the displacement rather than with the instantaneous motion, which indicates that coregistration errors generated by the change in head position relative to the co-registered points at the start of the recording are responsible for these correlations. It is also notable that the frontoparietal (FP) network does not exhibit correlations with the head movement metrics, while the other networks do have such correlations. This could be due to the fact that the FP network is not as concentrated spatially as the other networks, i.e. it involves connections among 4 spatially separate areas. For erroneous, head-movement induced network activity to be present in the FP network, all 4 areas would need to be showing head movement induced errors in the same direction at the same time. We do note here that the uncorrected p-values that are reported in Table 4 do not survive the FDR correction for the 30 multiple comparisons we perform. For that reason we interpret these findings as descriptive trends.

Regressing the head movement metrics out of the beamformer envelope time series completely eliminates the correlations between network activation and the head movement metrics for the five networks we examine (Table 5). In presenting the slopes in Table 5, we have multiplied their values by 10^10^, which means that, for example, the slope for linear regression between the DMN and the instantaneous motion of the nasion is equal to −0.237 × 10^−10^.

### 3.5 Changes in the network maps (connectivity)

As explained in Sec. 2.7, we calculate the cosine similarity for the five networks without head movement correction and with head movement correction resulting from regressing out the six head movement metrics. The cosine similarity and the resulting angles for the comparison are shown in Table 6.

**Table 6:** Cosine similarity and angle between the vectors consisting of the coefficients of the un-mixing matrix of the ICA. DMN = default-mode, FP = frontoparietal, SM = sensorimotor, VIS = visual, FL = frontal lobe.

		cosine similarity	angle (degrees)
No head movement correction versus regressing-out head movement metrics	DMN	0.992	7.2
	FL	0.986	9.5
	FP	0.992	7.1
	VIS	−0.015	90.9
	SM	0.843	32.5

Comparing the networks derived with no head movement correction to those derived after regressing out the head movement metrics, we notice that the DMN, the FP and the FL networks have a cosine similarity close to 1 (and a corresponding angle of under 10°), indicating that the vectors consisting of the respective coefficients of the un-mixing matrix resulting from the ICA are very similar to each other and therefore the spatial patterns of connectivity of those networks do not differ fundamentally. For the visual and sensorimotor networks, on the other hand, the cosine similarity is smaller than 1, and the resulting angles are 90.9 and 32.5° respectively. This means that the voxels implicated in those two networks appear in them with different weights, and therefore that the connectivity maps for those networks differ, once the head movement correction has been applied. We note here that, due to the nature of the cosine similarity as a measure of similarity, there is no specific a-priori method for assigning a threshold that would distinguish networks that are highly similar from networks that are not similar, in particular for spaces that are of high-dimensionality as in this study. In our case, however, the divide between the networks that are highly similar before and after head movement correction (angles of under 10°) and those that are not (angles of over 30°) is clear-cut, and for that reason we can unequivocally distinguish between those networks for which connectivity maps are not altered and those for which they are.

As can be seen in Fig. 12, the changes in spatial structure of the visual and sensorimotor networks primarily involve a narrowing of the regions toward the core nodes. In the visual network in particular, these nodes appear to have shifted slightly ventrally. This important result has implications for studies where populations with different characteristics are compared to each other using MEG. It indicates that if head movement correction is not applied, artifactual connectivity differences that arise from head movement can be categorized as physiological differences of functional connectivity, leading to erroneous conclusions.

## 4 Discussion

### 4.1 Assessment and explanation of the results

The MEG community places great importance in eliminating from MEG analyses the errors that result from head movement in the MEG system. In our study we aimed to investigate the correlations of head movement metrics with resting-state measures of source-localized oscillatory brain activity. We also aimed to explore, assess and compare ways to minimize any such correlations using widely-available software tools.

We showed that head movement explains a significant proportion of the variability of the beamformer envelope time series, in all frequency bands considered. This is indicated both by the large values of the coefficient of determination R^2^ and by the large number of voxels exhibiting non-zero R^2^. Applying a participant-specific high-pass filter, with a high-pass frequency determined by the displacement time series of each participant, significantly reduces these correlations. Specifically, the maximum R^2^ seen in any one voxel drops to about 0.05 or less and the average number of voxels affected by such correlations is nearly zero in all frequency bands considered. The remaining correlations are likely correlations with the instantaneous motion, which the high-pass filter cannot remove because it is based on a high-pass frequency calculated from the displacement time series. On the other hand, regressing the six head movement metrics out of the beamformer envelope time series practically eliminates those correlations. The remaining effects are very small and most likely due to the fact that the linear regression used to identify the coefficients employs a least-squares algorithm which minimizes, but does not completely eliminate, the residuals.

Furthermore, we showed that the network activation for four of the networks we examined showed a trend for correlation with the displacement of the fiducial coils, which was only significant when assessed without correction for multiple comparisons. These correlations are most likely due to coregistration mismatch resulting from the gradual drift of the participants' head positions compared to the position at the start of the recording, which affect the source localization process at beamformer envelope time series calculation. The network activation also showed a trend for correlation with the instantaneous motion of some of the fiducial coils for the default mode network and the frontal lobe network. This indicates that some of the correlated source variability that is identified as network activity is in fact due to the participants moving within each second of the recording.

The network-selective trends for correlations with head motion may be due to a smaller contribution of true signal in the affected networks (DMN, SM, VIS, FL) compared to the FP network that was not correlated with head motion. A beamformer will generate a source solution for every voxel, even if little actual neural signal is present, and in the case of no signal it will reflect pure noise. A reconstructed signal containing mostly noise could be significantly influenced by fluctuations in head position relative to the sensors. This has considerable implications for the interpretation of MEG-derived functional resting-state networks. A frontal lobe network would be influenced by head motion, because the front of the head is affected by a pitch head motion or a downward displacement that are likely to take place during a MEG recording, especially in seated position where the participant may rest the back of their head against the inside of the dewar. However, it is concerning that the widely-researched DMN would fall into this category. Possibly, the fact that the DMN consists of mainly deeper brain areas that are far away from the sensors renders it sensitive to noise in the MEG. Alternatively, the DMN identified here was heavily frontal-dominant, which may have contributed to its sensitivity to head motion similar to the frontal lobe network.

Additionally we demonstrated that regressing the six head movement metrics out of the beamformer envelope time series, akin to what is common in the fMRI literature and previously applied to MEG data (Stolk et al., 2013), completely eliminates the correlations between network activation and those head movement metrics. It is therefore a very powerful tool in dealing with head movement in this context.

We also showed that regressing the six head movement metrics out of the beamformer envelope time series results in changes in the spatial patterns of connectivity (network maps) for the visual and sensorimotor networks. This is a very important finding, because it applies to the two networks that were least affected by head motion in their temporal component amplitude, and are typically two of the most robustly obtained networks in temporal ICA MEG analysis (Wens et al., 2014). ICA-derived resting-state network analyses are typically conducted under the assumption that the spatial aspect of components does not differ among individuals. Our findings suggest that this assumption may be invalid in light of head motion differences and warrants care when interpreting network differences between groups consisting of individuals that may exhibit significant or systematic differences in head motion.

It should be noted that the participant-specific high-pass filter that we present as a possible head movement correction method potentially discards low-frequency contributions to the amplitude envelope of the beamformer time series and the network activation that are of physiological origin. This is a drawback of this method in comparison with the correction that results from regressing out the head movement metrics.

### 4.2 Limitations and future work

This work has a few limitations that could be addressed in future studies.

Firstly, the analysis included a relatively small number of participants (24). The fMRI head-motion analyses presented in Van Dijk et al. (2012), and in Mowinckel et al. (2012) included over 1000 and over 200 participants respectively, which gives them greater statistical power and more robustness to the results. Studies that include a larger number of participants could shed more light into the effects of head movement on MEG resting-state analyses.

Secondly, characterization of network behavior between brain areas were performed for amplitude-to-amplitude correlations of the beamformer envelope time series in different voxels. Network behavior can also be evoked by means of amplitude-phase and phase-phase correlations of beamformer time series (Siegel et al., 2012). It should therefore also be investigated whether networks derived through such correlations are similarly affected by head movement in the MEG system. Other factors such as choice of beamformer or other source imaging techniques (such as minimum norm estimates) should also be explored, as well as seed-based correlation maps. Furthermore, in this paper we only address whether head movement has an effect on, and can be regressed out of, beamformer source-localized envelopes of resting-state oscillatory data. The influence of head motion is not limited to the inverse solution, and will in fact already have an effect at the level of signal generation. The effect of head motion on forward modeling may differ from the effect on the inverse solution and should be studied separately in future work.

Additionally, the beamformer/ICA analysis used to generate the resting-state networks is only performed within a given frequency band of interest. However, network activity can also be coordinated by neural oscillations across different frequency bands (Donner and Siegel, 2011, Jensen and Colgin, 2007). Networks identified through cross-frequency activity may well show a different pattern of sensitivity to head movement and hence should be the subject of future investigations.

In this study, only 5 resting-state networks were considered. It would be important to identify whether head movement affects the time courses of other networks, both resting-state and task-related ones. In particular for task-related studies, it would be important to take into account the fact that some tasks (for example button-pressing ones) may cause participants to move more and in different ways to the way they move during resting-state recordings, and that movement may be more correlated with the stimuli used and with the observed responses. Additionally, it would be important to assess whether statistically significant correlations exist between head movement and the beamformer envelope time series for such tasks.

Another issue that relates to head movement is whether or not it can have an effect on the reliability of resting-state measures of oscillatory brain activity when participants are tested multiple times, for example in longitudinal studies. Some work has tried to address the issue of network repeatability and reliability from perspectives other than head movement corrections (for example Colclough et al., 2016, Nugent et al., 2015, Wens et al., 2014), because variability induced by factors other than brain changes could influence the way the results of such studies are interpreted and lead to erroneous conclusions. As far as head movement is concerned, some preliminary (and currently unpublished) results from our group from retesting participants suggest that head movement in the MEG system is a participant trait, i.e. the head movement metrics exhibit correlations for multiple recordings of the same participant, at least when the testing and retesting happen within a few months of each other. This result is in agreement with what is reported in the fMRI literature for head movement in the MRI scanner (for example Van Dijk et al., 2012) and is somewhat reassuring. However, more studies, in particular studies that involve testing/retesting with time separations longer than a few months, should be performed to evaluate whether head movement in the MEG system is indeed a personal trait.

Finally, the present analysis involved only healthy adult participants that only exhibited relatively small amounts of head motion during the recording. Nevertheless, we find considerable correlations of head motion with oscillatory beamformer time series. It is necessary to perform similar analyses for clinical populations as well as for children and elderly adults, so that the validity of conclusions drawn in existing studies of such populations can be assessed. Some clinical populations, especially ones that are older, tend to move more, as do children whose heads are smaller and who may find it uncomfortable to use additional padding that would limit their range of movement in the MEG system.

If statistically significant correlations are identified for other MEG-derived brain activity metrics or for different population groups, it would be mandatory to employ robust ways to remove those effects so that the true correlates of neural activity are elucidated. This is additionally important because Wehner and colleagues (Wehner et al., 2008) reported that the level of error due to head movement in their analysis was comparable to the error from other sources such as measurement noise or forward-modeling noise. As indicated in our analysis, regressing out the appropriate head movement metrics eliminates these effects. If different groups exhibit different levels of head movement and if, as the results presented in this study indicate, head movement affects different measures of brain activity in different ways, it would be important to consider head movement on a case-by-case basis, especially in studies that aim to examine individual differences between participants.

## Author contributions

EM, LK, KDS designed the study; EM, LK, DCD, GMW, GP performed the scanning; EM, LK, GP developed software tools; EM performed the analysis; EM, LK wrote the paper.

## Figures and Tables

**Fig. A1:**
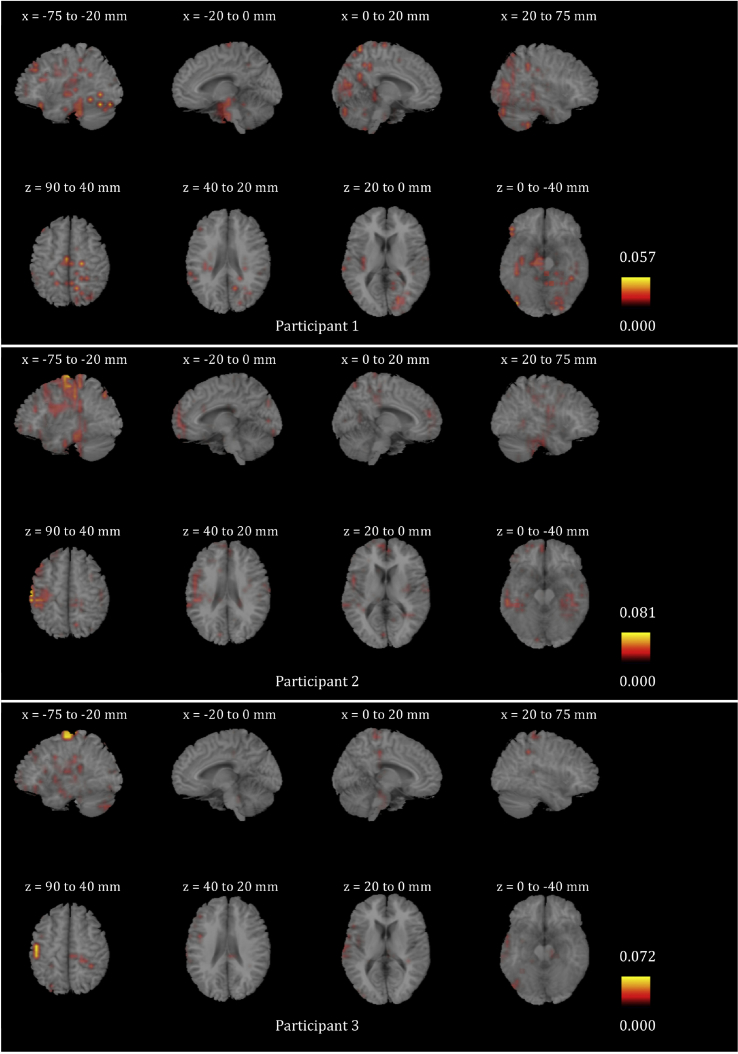
R^2^ for the voxel-wise correlations in the delta frequency band for 3 participants. For each participant, the maps show four sagittal (top) and axial (bottom) slices averaged over the *x* and *z* Talairach coordinates as indicated. FDR correction for multiple comparisons has been applied and the images show only voxels that survive the correction.

**Fig. A2:**
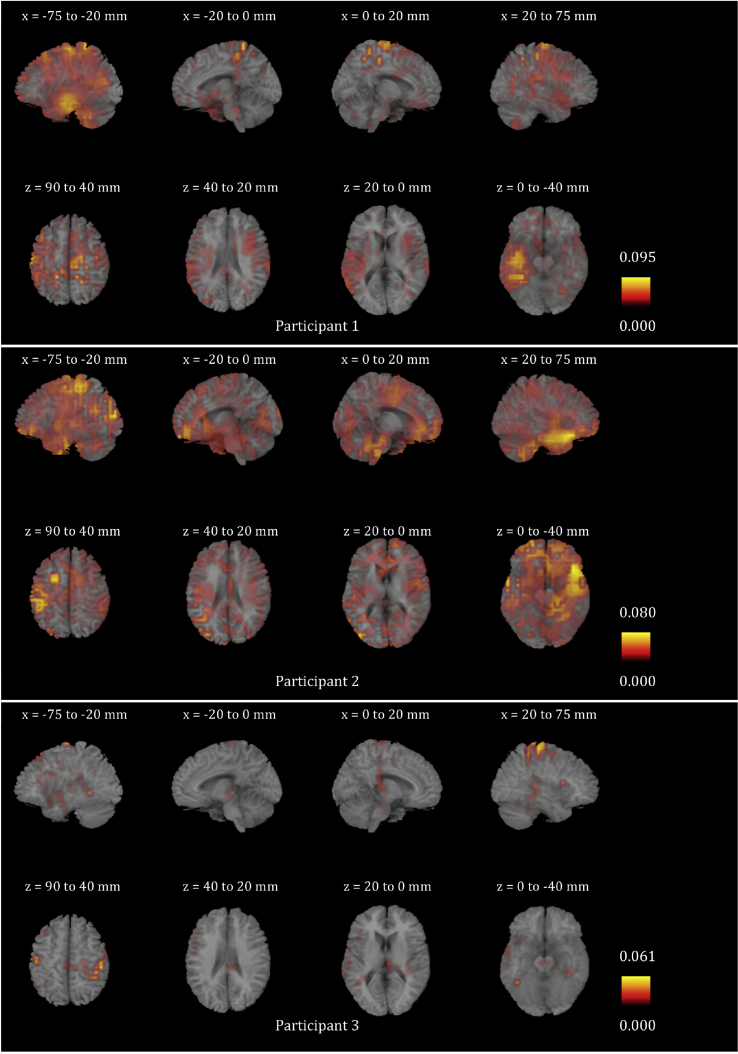
R^2^ for the voxel-wise correlations in the theta frequency band for 3 participants. For each participant, the maps show four sagittal (top) and axial (bottom) slices averaged over the *x* and *z* Talairach coordinates as indicated. FDR correction for multiple comparisons has been applied and the images show only voxels that survive the correction.

**Fig. A3:**
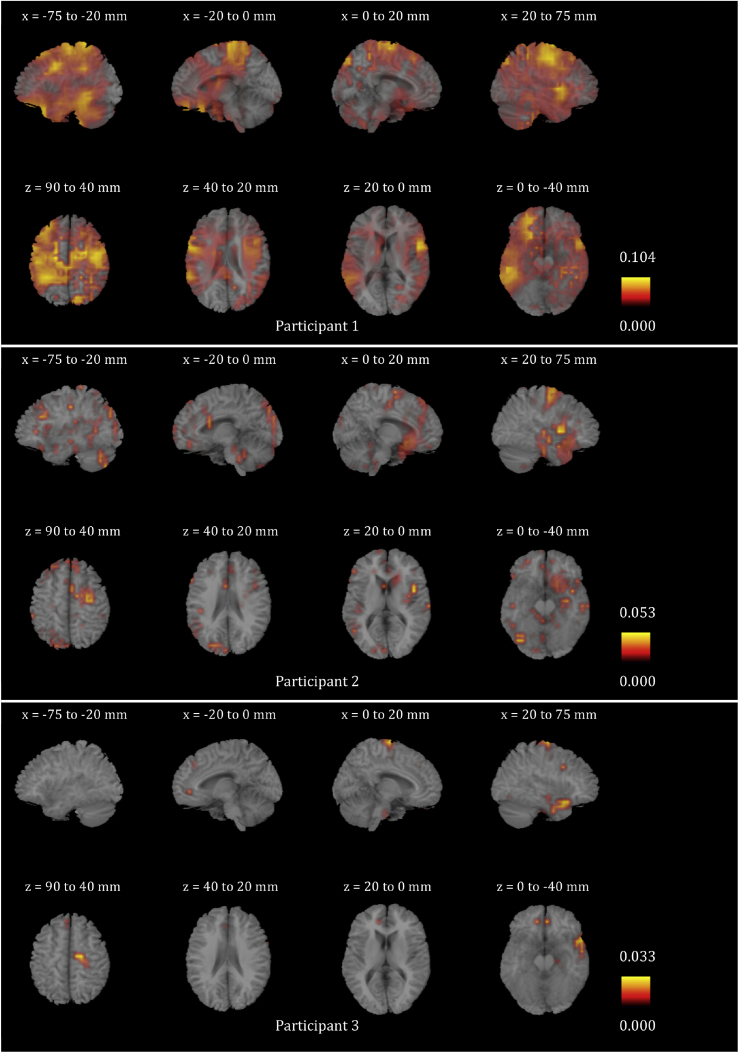
R^2^ for the voxel-wise correlations in the alpha frequency band for 3 participants. For each participant, the maps show four sagittal (top) and axial (bottom) slices averaged over the *x* and *z* Talairach coordinates as indicated. FDR correction for multiple comparisons has been applied and the images show only voxels that survive the correction.

**Fig. A4:**
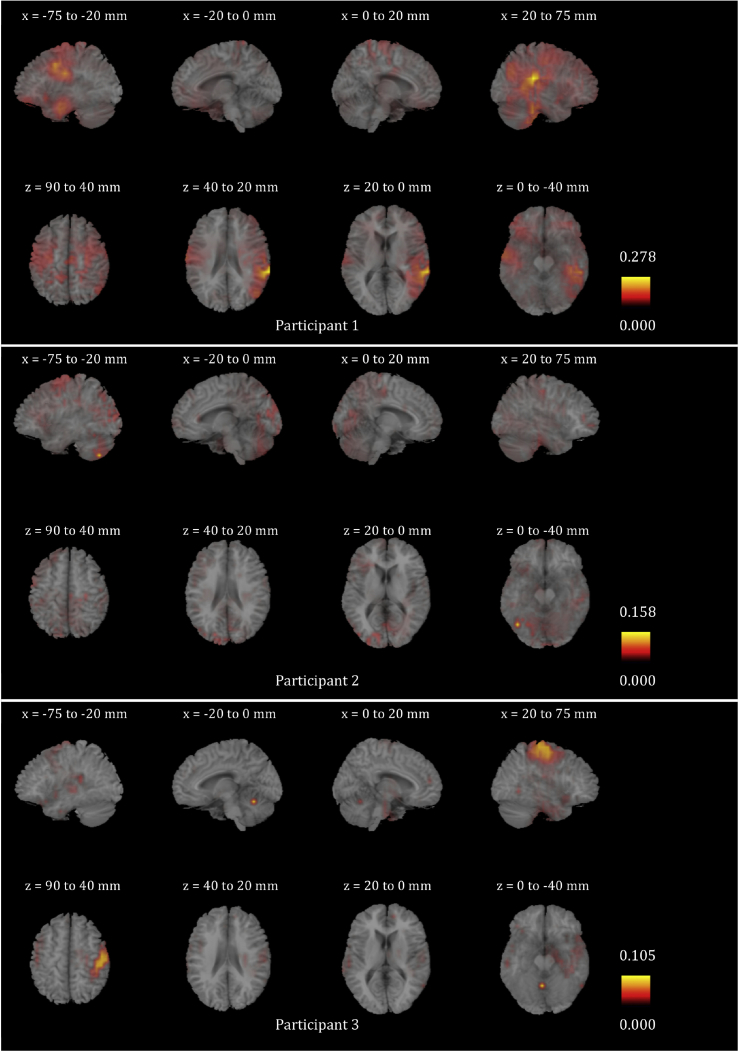
R^2^ for the voxel-wise correlations in the beta frequency band for 3 participants. For each participant, the maps show four sagittal (top) and axial (bottom) slices averaged over the *x* and *z* Talairach coordinates as indicated. FDR correction for multiple comparisons has been applied and the images show only voxels that survive the correction.

**Fig. A5:**
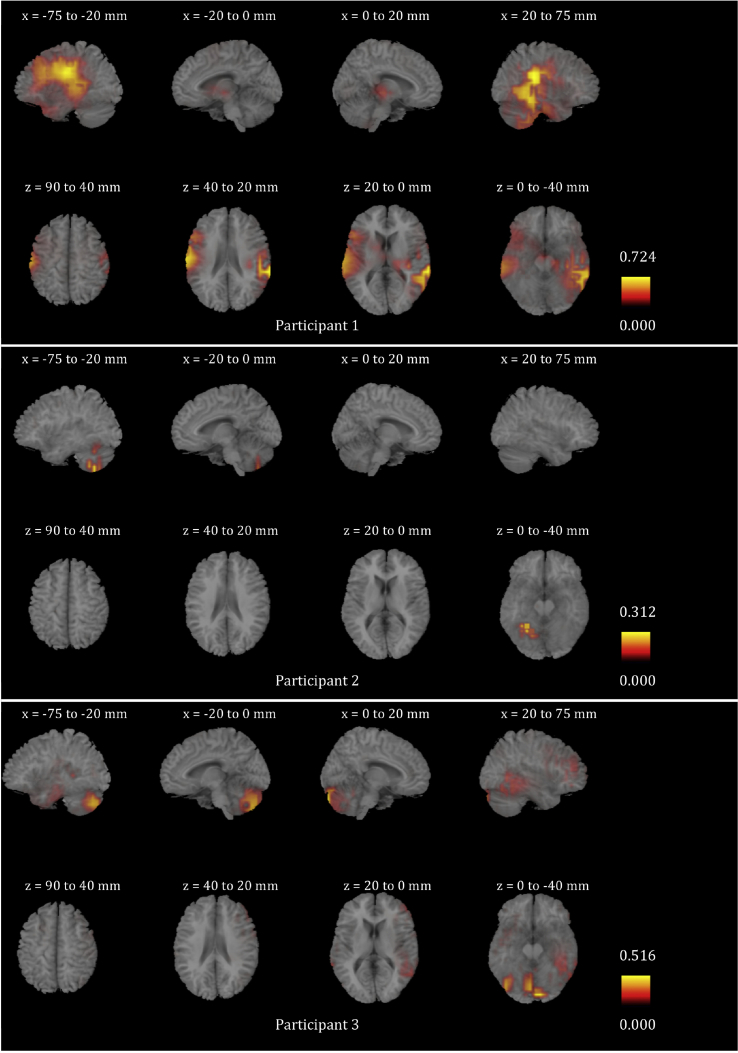
R^2^ for the voxel-wise correlations in the lower gamma frequency band for 3 participants. For each participant, the maps show four sagittal (top) and axial (bottom) slices averaged over the *x* and *z* Talairach coordinates as indicated. FDR correction for multiple comparisons has been applied and the images show only voxels that survive the correction.

**Fig. A6:**
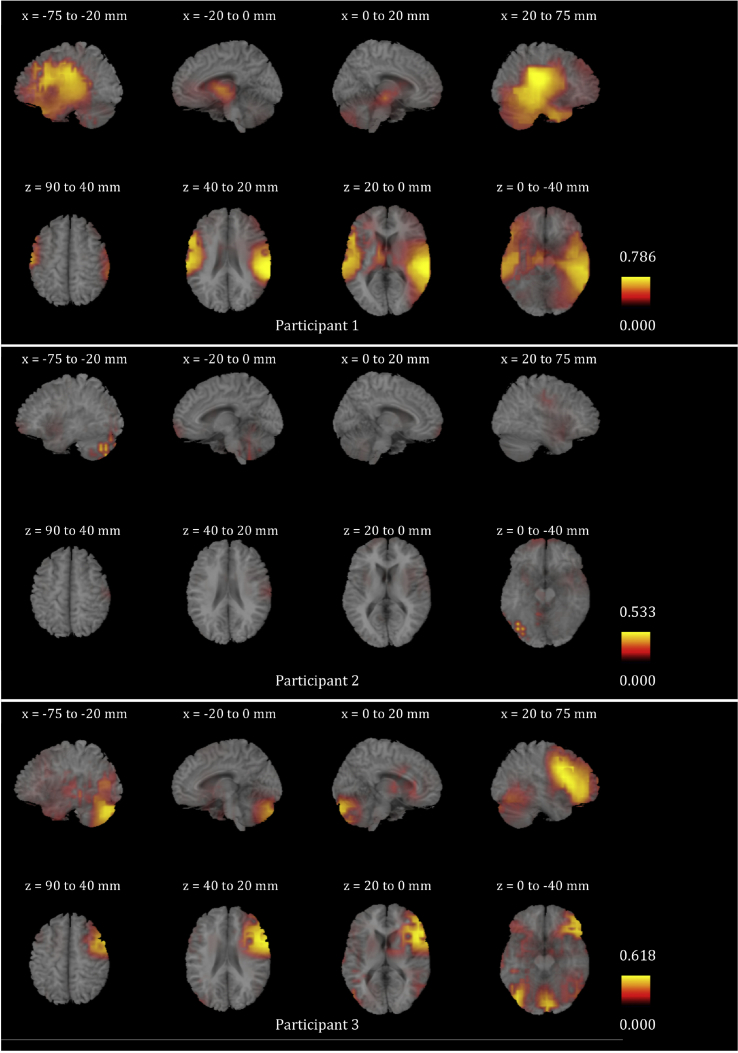
R^2^ for the voxel-wise correlations in the upper gamma frequency band for 3 participants. For each participant, the maps show four sagittal (top) and axial (bottom) slices averaged over the *x* and *z* Talairach coordinates as indicated. FDR correction for multiple comparisons has been applied and the images show only voxels that survive the correction.

## Appendix A

In order to demonstrate that the voxel-wise R^2^ maps described in Sec. 3.2 are very different for different participants, we present in this appendix the maps for three participants in the six frequency bands considered.
